# Genetic Testing for Chronic Kidney Diseases: Clinical Utility and Barriers Perceived by Nephrologists

**DOI:** 10.1016/j.xkme.2021.08.006

**Published:** 2021-10-05

**Authors:** Michal Mrug, Michelle S. Bloom, Christine Seto, Meenakshi Malhotra, Hossein Tabriziani, Philippe Gauthier, Vicki Sidlow, Trudy McKanna, Paul R. Billings

**Affiliations:** 1 Department of Medicine, The University of Alabama at Birmingham, Birmingham, Alabama; 2 Department of Veterans Affairs Medical Center, Birmingham, Alabama; 3 Natera, Inc, San Carlos, California

**Keywords:** Genetic testing, next-generation sequencing, inherited, hereditary, familial, renal, kidney, disease, disorders, survey

## Abstract

**Rationale & Objective:**

The identification of pathogenic variants in genes associated with chronic kidney disease can provide patients and nephrologists with actionable information to guide diagnoses and therapeutic plans. However, many nephrologists do not use genetic testing despite costs decreasing over time and more widespread availability.

**Study Design:**

We conducted a survey to uncover the perceptions of general adult nephrologists about the utility of and barriers to genetic testing in clinical practice.

**Setting & Participants:**

The online survey was administered to board-certified nephrologists (n = 10,054) in the United States.

**Analytical Approach:**

We analyzed demographic characteristics of the survey respondents and their responses in the context of their use of genetic testing in routine clinical practice.

**Results:**

A total of 149 nephrologists completed the survey, with 72% (107 of 149) reporting genetic test use in their practice. On average, tests were ordered for 3.8% of their patient population. Thirty-five percent of responses from nephrologists without a history of genetic test use ranked perceived barriers as “extremely significant” compared with 23% of responses from those who had previously used genetic tests. However, both users and nonusers of genetic tests indicated high cost (users: 46%, 49 of 107; nonusers 69%, 29 of 42) and poor availability or lack of ease (users: 33%, 35 of 107; nonusers: 57%; 24 of 42) of genetic testing as the most significant perceived barriers to implementation.

**Limitations:**

The survey used in this study was not previously validated; additionally, because of the relatively small number of responses, there might have been a selection bias among the responders.

**Conclusions:**

Although most nephrologists reported using genetic tests in clinical practice, high costs and poor availability or the lack of ease of use were perceived as the most important barriers to routine adoption. These observations indicate that educational programs that cover a range of topics, from genetics of chronic kidney disease to selection of the test, may help mitigate these barriers and enhance the use of genetic testing in nephrology practice.

**Plain-Language Summary**

The identification of mutations in genes associated with chronic kidney disease (CKD) can provide patients and nephrologists with actionable guidance for diagnostics and therapeutic plans. However, many nephrologists do not use genetic testing, despite decreasing costs and widespread availability. Therefore, we conducted a survey to uncover the perceptions of board-certified general adult nephrologists about the utility of, and barriers to genetic testing in clinical practice. Although most nephrologists reported using genetic tests, the costs, availability and ease of use were perceived as the most important barriers to routine adoption. Educational programs covering a range of topics from genetics of CKD to test selection may help mitigate these barriers and enhance the use of genetic testing in nephrology practice.

Chronic kidney disease (CKD) is a significant public health challenge, with few curative therapies and a prevalence of ∼15% in the United States.^1^ In 2017, CKD affected ∼700 million people globally, leading to 1.2 million deaths.^2^ In the most severe cases, CKD progresses to end-stage kidney disease, which requires costly kidney replacement therapies such as dialysis or kidney transplantation.^3^ Moderately advanced CKD can also cause multiorgan complications that increase morbidity and mortality. For example, CKD is a major risk factor for cardiovascular disease. In 2017, deaths due to CKD and CKD-attributable cardiovascular disease accounted for 4.6% of worldwide all-cause mortality.^2^

Over 30% of patients with CKD report a family history.^4^ Additionally, a pathogenic variant (also called a mutation) in a single gene can be identified in ∼10% of adult patients with CKD,^5^ with over 500 monogenic disease-linked variants identified to date.^4^ Although the contribution of many additional variants to the pathogenesis of adult CKD remains unknown,^6^ the characterization of these genes and the proteins they encode continue to advance the understanding of CKD causality. Furthermore, these discoveries have enabled the development of targeted diagnostic, prognostic, and therapeutic strategies for these disorders.

The management of genetic and acquired forms of CKD can differ, and determining the underlying cause of CKD using traditional diagnostic tools can be challenging.^7^ The identification of pathogenic gene variants in CKD-associated genes may provide patients and nephrologists with actionable information that can help guide diagnoses, help with the development of therapeutic plans, and lead to referrals for the management of extrarenal complications. Furthermore, establishing a genetic cause of CKD or end-stage kidney disease may influence the medical management of patients’ family members. For example, establishing a genetic diagnosis can lead to the testing and screening of family members, and the outcomes of such testing may directly inform their medical management (eg, early diagnosis and medical care, family planning, or the selection of living related donors for transplantation). Additionally, the identification of pathogenic CKD gene variants can provide diagnostic information complementary to, or instead of, a kidney biopsy.

Historically, the use of genetic testing in patients with CKD was limited by the high costs of DNA sequencing. However, advances in next-generation sequencing have enabled the development of several lower-cost clinical platforms offering simultaneous testing of multiple CKD genes.^8^ Nevertheless, despite the advances in technology and the decreased costs, genetic testing has not been broadly adopted by nephrologists. To identify the reasons for this underutilization, we surveyed nephrologists practicing in the United States about their current practices and perceived utility of genetic testing as well as barriers to the adoption of these tests.

## Methods

### Survey Design and Content

We developed a 29-question online survey based on previous market research surveys and patient focus groups conducted by Natera, Inc. The survey contained multiple-choice, ranking, and fill-in-the-blank questions (Table S1). For questions regarding the perceived potential barriers to genetic testing, respondents ranked each of the provided categories on a scale of 1 (not a barrier) to 5 (significant barrier). The survey participants also ranked the importance of different potential types of support to enable easier ordering of genetic tests on a scale of 1 (not important) to 5 (extremely important). For questions regarding identification of clinical scenarios and specific clinical diagnoses for which genetic testing may hold value, the participants were allowed to select multiple categories.

### Dissemination and Study Sample

The survey was hosted by SurveyHealthcareGlobus, which used a technology suite with a proprietary healthcare professional panel to identify and administer the survey to potential respondents. The survey was distributed to 10,054 nephrologists in the United States via email (the American Board of Internal Medicine reported 10,901 nephrologists with valid board certification in the United States in 2020^9^). The respondents were required to answer screening questions to review their eligibility before proceeding to the survey questions (Table S1). The exclusion criteria included the following: (i) selection of a specialty that was not general adult nephrology (ie, transplant nephrology, pediatric nephrology, surgery, other), (ii) not board certified in general nephrology, or (iii) practicing nephrology for less than 2 years. Respondents who completed the survey received a $35 honorarium through the survey host. The survey was released in 2 phases in July 2020 and was closed 16 days after the initial launch once 150 eligible responses were obtained.

All survey administration and reminder emails and payments for survey completion were conducted by SurveyHealthcareGlobus. Natera, Inc. was not involved in the selection of the participants, administration of the survey, or advertisement of the survey. There were no indications on the survey that it was developed or sponsored by Natera, Inc. This study did not involve any patient data; rather, it was a survey of physicians and their attitudes toward genetic testing. Thus, the study was exempted from institutional review board requirements. All the respondents agreed to participate in the survey, and informed consent was not needed because no health-related data were collected.

### Data Analysis

Descriptive statistical analyses were performed to summarize demographics and the frequency of the survey responses. Comparisons of test ordering habits based on levels of education were analyzed using the Kruskal-Wallis test (*P* value <0.05 was considered significant). An analysis of questions involving the ranking of barriers and resources was performed by the quantification of the responses using the 5 (significant barrier or extremely important) categories.

## Results

### Demographic Data

Of the 10,054 nephrologists contacted, 209 responded to the 29-question survey (gross response rate of 2.1%). However, 60 responses were excluded because of ineligibility (n = 50), incompletion of the survey (n = 9), or invalid responses (n = 1). In a final cohort of 149 respondents (effective response rate of 1.5%), general nephrologists represented 35 states (including the District of Columbia), with the largest proportion of 38.9% (58 of 149) located in the southern region, consistent with the breakdown of US board-certified nephrologists in 2020, as reported by the American Board of Internal Medicine (Table 1).^9^ Sixty-nine percent (n = 103) of the survey respondents worked in private practice settings, with 29% (n = 43) in large practices of >10 providers and 40% (n = 60) in small practices with <10 providers, which was consistent with data regarding the nephrology workforce reported by the American Medical Association.^10^ The largest proportion of respondents (46%; n = 69) practiced nephrology for a period of 10-20 years (Table 1).

**Table 1:** Demographics of Survey Respondents

Affiliation	n (%)
University or academic institution	32 (21.5)
Nonacademic medical center	14 (9.4)
Large private practice (>10 providers)	43 (28.8)
Small private practice (<10 providers)	60 (40.3)
**Region**	
Midwest	29 (19.5)
Northeast	28 (18.8)
South	58 (38.9)
West	34 (22.8)
**Years of Practice, y**	
<2	0 (0.0)
2-5	5 (3.3)
5-10	25 (16.8)
10-20	69 (46.3)
20-30	39 (26.2)
≥30	11 (7.4)
**Genetics Education**	
None	10 (6.7)
Limited	108 (72.5)
Extensive	31 (20.8)

### Trends in Genetic Test Use for CKD Among General Nephrologists

**Figure 1:**
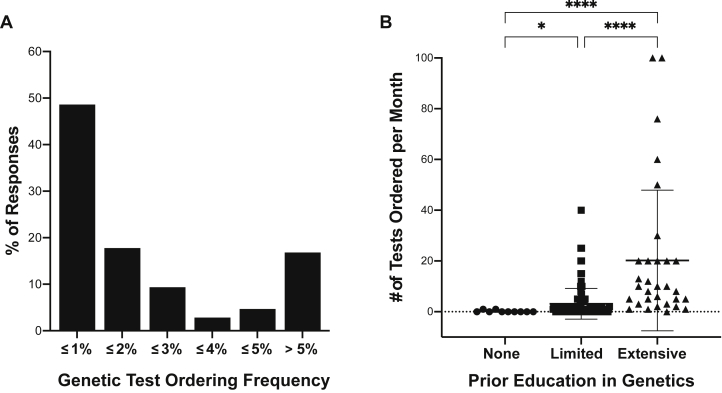
Genetic test ordering frequency and its relationship with genetics education among US nephrologists. (A) The genetic test ordering frequency was calculated among “users” (n = 107) based on the reported number of tests ordered per month and number of unique patients seen per month for each respondent. The proportion of nephrologists who ordered genetic tests for <1% of their patients was 48.6%. (B) The reported number of tests ordered per month were stratified by the level of education in genetics indicated by each respondent (n = 149). A positive relationship was identified between physician education in genetics and the number of genetic tests ordered per month. Nephrologists with prior education in genetics ordered more genetic tests than those with no such education. Similarly, those with extensive education in genetics ordered genetic tests more frequently than those with only limited education. ∗*P* < 0.05. ∗∗∗∗*P* < 0.0001 by Kruskal-Wallis test.

Among the 149 respondents (i.e., nephrologists) comprising the final cohort, 107 (72%) reported ordering ≥1 genetic test, on average, per month (designated as "users"), whereas 42 (28%) reported ordering an average of 0 genetic tests per month (designated as "nonusers"); Iit is possible that “nonusers” also ordered genetic tests, but at a rate of <1 per month. The median number of genetic tests reported by “users” with a history of ordering was 2 per month (range: 1-100). The average proportion of patients for whom genetic tests were ordered was 3.8% (SD 7.4%). Eighty-three percent (n = 89) of the respondents indicated that they tested ≤5% of their patients, whereas 49% (n = 52) indicated that they tested <1% of their patient population (Fig 1A).

### Recall of Prior Training in Genetics Among Nephrologists

To understand the relationship between training in genetics and the use of genetic testing among nephrologists, the survey respondents ranked their education in genetics: 21% (n = 31) indicated “extensive”, 72% (n = 108) indicated “limited”, and 7% (n = 10) indicated no prior education in genetics. Comparison of the reported numbers of tests ordered per month, when stratified by each respondent’s level of education in genetics, revealed that nephrologists with “extensive” education in genetics ordered, on average, more genetic tests (20.2 ± 27.7) than those with “limited” (3.1 ± 6.1) or no education (0.3 ± 0.4; *P* < 0.001) (Fig 1B).

### Perceived Utility of Genetic Testing in the General Nephrology Setting

The surveyed nephrologists were asked to identify clinical scenarios in which they thought that genetic testing held the most value among the provided categories: (i) all patients with CKD, (ii) patients with CKD of unknown etiology, (iii) patients with specific clinical diagnoses, or (iv) pediatric patients. The majority, regardless of their history of genetic testing use, indicated that genetic testing was the most valuable for patients with specific clinical diagnoses (users: 75%, 80 of 107; nonusers: 86%, 36 of 42) and for the evaluation of patients with CKD of unknown etiology (users: 69%, 74 of 107; nonusers: 38%, 16 of 42; Table 2). The specific clinical diagnoses for which genetic testing was indicated to be the most valuable were cystic diseases (users: 69.2%, 74 of 107; nonusers: 76.2%, 32 of 42) and glomerular diseases (users: 56.1%, 60 of 107; nonusers: 52.4%, 22 of 42) (Table 2).

**Table 2:** Perceived Utility of Genetic Testing in Specific Clinical Contexts

	Users	Nonusers
**n**	107 (%)	42 (%)
**Clinical Scenarios**		
All patients with CKD	12 (11.2%)	0 (0%)
CKD of unknown etiology	74 (69.2%)	16 (38.1%)
Specific clinical diagnoses	80 (74.8%)	36 (85.7%)
Pediatric patients	27 (25.2%)	13 (31%)
I do not see the value in genetic testing	N/A	2 (4.8%)
**Specific Clinical Diagnoses**		
Cystic	74 (69.2%)	32 (76.2%)
Glomerular	60 (56.1%)	22 (52.4%)
Electrolyte abnormalities	39 (36.5%)	20 (47.6%)
Tubulointerstitial disease	38 (35.5%)	8 (19.1%)
Nephrolithiasis	33 (30.8%)	9 (21.4%)
CAKUT	29 (27.1%)	6 (14.3%)
Hypertension	14 (13.1%)	4 (9.5%)
Diabetic nephropathy	8 (7.5%)	0 (0.0%)
Other (TMA)	1 (0.9%)	0 (0.0%)

Abbreviations: CAKUT, congenital anomalies of the kidney and urinary tract; CKD, chronic kidney disease; N/A, not available; TMA, thrombotic microangiopathy.

### Perceived Barriers to Genetic Testing

**Figure 2:**
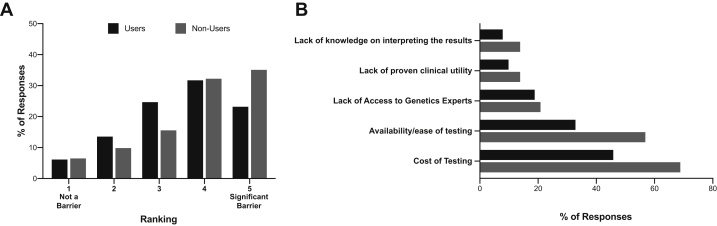
Perceived barriers by both users and nonusers of genetics tests. (A & B) Respondents ranked potential barriers to testing on a scale of 1 (not a barrier) to 5 (significant barrier). (A) Quantification of responses for each ranking on the scale of 1 to 5 demonstrated that nonusers perceived barriers to be more significant compared with users. (B) Quantification of responses ranked as 5 (significant barrier) for each potential barrier indicated that “cost of genetic testing” and the “availability or ease of testing” were perceived as the most important barrier types by both users and nonusers of genetic tests.

The nephrologists ranked each of 5 potential barriers to genetic test use (cost of testing, availability or lack of ease of testing, access to genetics experts, lack of proven clinical utility, knowledge about result interpretation) on a scale of 1 (least significant) to 5 (most significant) (n = 730 responses). The largest proportion of responses from nonusers were ranked as “5” (35%, 74 of 210), whereas the highest proportion of responses from users (33%, 172 of 520) scored barriers as “4” on the 5-point scale (Fig 2A). Regardless of their history of genetic test use, the nephrologists indicated that the cost of testing for patients was the most significant barrier (users: 46%, 49 of 107; nonusers 69%, 29 of 42). The second most significant barrier was poor availability or lack of ease of testing (users: 33%, 35 of 107; nonusers: 57%; 24 of 42). The least significant barriers reported were the lack of proven clinical utility and the lack of knowledge about result interpretation (Fig 2B).

### Perceived Utility of Resources That May Overcome Barriers to Genetic Testing

The nephrologists also ranked the importance of 4 resources that have been proposed to overcome the barriers to genetic testing on a scale of 1 (not important) to 5 (extremely important) (n = 596 responses). All provided resources were considered extremely important, with 46% (273 of 596) of the total responses falling into the “5” category (Table 3). Furthermore, the survey respondents indicated high interest in all educational resource options for physicians and patients (Table 3).

**Table 3:** Perceived Needs for Resources to Overcome Barriers to Genetic Testing

	Total
**N**	149 (100%)
**Resources**	
Detailed results with implications for patient and/or biological relatives	78 (52%)
Insurance or billing support	75 (50%)
Easy ordering process	69 (46%)
Access to genetic counselors for myself and my patients	51 (34%)
**Educational Resources for Physicians**	
Refresher on genetic causes of CKD	100 (67%)
Education about how to talk to patients about options for testing and result implications	81 (54%)
Resources to help educate patients on genetic causes of CKD and family planning needs	103 (69%)
None	5 (3%)
**Educational Resources for Patients**	
Basics of genetic kidney disease	107 (72%)
Implications for family, such as living related donors, family planning	126 (85%)
Details on how to interpret results of genetic tests	111 (75%)
Privacy of genetic information	66 (44%)
Insurance implications of a positive finding	89 (60%)

Abbreviations: CKD, chronic kidney disease.

## Discussion

Genetic testing has transformed diagnostic, prognostic, and therapeutic approaches to many inherited disorders, including CKD. However, the extent of adoption of genomic medicine practices, including genetic testing, in nephrology remains low.^10^ Therefore, we conducted a survey to understand the perceptions and barriers that nephrologists may have toward the use of genetic tests and to identify aspects in which educational resources may be useful. The survey was not intended to guide clinical management decisions based on genetic testing results.

Our study revealed that most nephrologists who responded to our survey had a history of using genetic testing (72%) and that they appreciated its value in the management of inherited forms of CKD. The highest perceived utility was observed for CKD of unknown etiology and for cystic and glomerular diseases. Such results suggest that general nephrologists understand the value of genetic testing, especially in clinical contexts that are likely to yield a genetic diagnosis. Therefore, it is expected that an enhanced understanding of relevant genetic factors that contribute to kidney function loss (eg, through education programs) will foster broader adoption of genetic testing by general nephrologists. For example, genetic testing has been shown to uncover inherited causes in up to 20% of cases of CKD with unknown etiology^5^ and can result in reclassification of clinical diagnoses in ∼20% of cases.^4^^,^^7^^,^^11^ The identification of pathogenic variants in CKD-related genes can also inform prognosis and treatment. Food and Drug Administration-approved therapies are currently available for some well-characterized genetic disorders such as autosomal dominant polycystic kidney disease.^12^ Moreover, an improved understanding of the genetic etiologies of kidney diseases may inform the development of new therapeutic strategies for these diseases (eg, nephronophthisis; reviewed in a study by Stokman et al^13^ and Slaats et al^14^). Additionally, subtypes of CKD can often share a phenotypic overlap with other diseases, such as Alport syndrome—the most common inherited form of glomerular CKD—which is caused by pathogenic variants in the *COL4A3*, *COL4A4*, or *COL4A5* genes, and steroid-resistant nephrotic syndrome, which is associated with variants in over 40 genes.^15^ However, other forms of CKD that are generally thought to be acquired, such as hypertensive and diabetic nephropathies, for which few nephrologists in our survey indicated a potential value in genetic testing, can also have heritable components.^16^, ^17^, ^18^, ^19^ Progression of hypertensive and glomerular kidney diseases is also associated with genetic risk factors, such as variants in *APOL1*, which are present in ∼10% of the African American population.^20^ The use of genetic testing to identify such at-risk individuals may allow the implementation of early targeted intervention programs for those with increased risk of developing CKD.

Our findings also revealed that 28% of the nephrologists had not used genetic tests in their practice. This value might have grossly underestimated the actual proportion of nonusers of genetic tests, given the low response rate to our survey and the possibility that genetic test users might have been more likely to respond to this survey. These findings are consistent with those of a recent study of attitudes toward the use of genetic testing among nephrology practitioners in Australia.^21^ The high proportion of nephrologists who have not used genetic tests is a potential concern in the context of ∼10% of CKD causes that are attributed to pathogenic variants in single genes.^5^ Increased use of genetic testing may help in identifying these patients and assist in their medical management.

Similar to our study, Jayasinghe et al^21^ found that most providers considered genetic testing useful in managing patients with suspected genetic forms of CKD. Taken together, the perception of high utility but low use of genetic testing suggests that significant barriers prevent its broader use by nephrologists. Our study identified several logistic perceived barriers, of which the most prominent was the high cost of genetic tests. However, according to the National Human Genome Research Institute’s data, the cost of sequencing 1 Mb (a million bases) has declined from $5,292.39 in 2001 to $0.008 in 2020, making next-generation sequencing approaches much more affordable and feasible.^22^ Additionally, next-generation sequencing-based testing is more cost effective than single-gene approaches for diseases in which clinical diagnosis can be difficult^23^ and can often be cheaper than diagnostic approaches such as kidney biopsy.^24^ Although our study did not include an analysis of the perceived costs of genetic testing, the rapid decline in the costs of next-generation sequencing and the expansion in testing options suggest that continued education about the cost effectiveness of genetic testing is needed.

Our study also identified the lack of ease of the genetic test ordering process as a barrier among nephrologists (by both users and nonusers of genetic tests). The inability to determine the appropriate genetic test was also recently identified as a major barrier to the use of genetic tests by nephrologists.^21^ A recent study indicated that approximately half of the genetic tests ordered by nongenetic providers were not the most appropriate option, as determined based on the patient’s clinical history, and that the selection of the correct tests resulted in lowered costs for both practices and patients.^25^ We identified a positive association between the level of genetics education and the number of tests ordered among the nephrologists in our survey. This finding indicates that providing educational resources in genetics may increase physician comfort in using genetic testing in their practices.

Among the nephrologist characteristics that we stratified for in our analysis (including US region and institution type), genetics education was the only characteristic that yielded significant differences in ordering patterns (Fig S1, Table S2). Despite the differences in the ordering patterns across different levels of genetics education, the significance of the barriers was perceived similarly by all groups (Table S3). These findings indicate that all general nephrologists may benefit from the dissemination of educational resources.

Our survey revealed high interest in educational resources spanning multiple topics, from the genetics underlying CKD to insurance implications. Two recent studies similarly indicated that nephrologists identified discomfort in speaking with patients about the results^26^ and interpretation of the genetic test results as challenges to test implementation.^21^ In addition to the interpretation of the results, our survey suggested a need for resources to help nephrologists facilitate discussions with patients about the potential benefits, limitations, and risks of genetic testing. There was high interest among the respondents in access to genetic counselors being provided for both physicians and patients as a needed resource (Table 3). Genetic counselors and other genetics experts can identify appropriate testing strategies, interpret results, and determine the appropriate screening of presymptomatic related family members.^27^ Thus, the availability of these resources for nephrology practices can assuage both physician hesitancies and patient concerns, and this may lead to the broader adoption of genetic testing. Additionally, laboratories offering genetic testing for CKD should implement comprehensive educational strategies and resources for physicians covering topics, such as current costs of different genetic testing options and genetic testing and billing processes, and initiating discussions with patients before and after performing genetic testing.

The current study’s major limitation is that it remains unknown how well our survey responses represent the entire surveyed group of nephrologists. Because of the small sample size and low response rate, it is possible that the survey responses were biased. Users of genetic tests might have been more motivated to respond to the survey, resulting in strongly overweighted sampling toward frequent users of genetic tests. Such a bias may explain the relatively high proportion of patients for whom genetic testing was ordered among all patients seen by individual nephrologists. Despite the potential for bias, this study provides valuable information related to the perceptions about genetic testing among nephrologists with or without a history of genetic test use. Another concern is the accuracy of the answers provided; this is because this survey was not administered directly by research staff and relied on the respondents’ opinions. For example, some responses provided for the number of genetic tests ordered (eg, 100 per month) seem excessively high. Finally, because of the relatively small number of responses, this study was underpowered to allow stratification based on specific responses and subsequent mediation analyses.

In conclusion, our survey sheds light on the clinical utility of, advantages of, and perceived barriers to using genetic tests among general nephrologists. These observations may support the development of educational programs that cater for increased appreciation of using genetic tests, which may help with patient management and improved formal practical guidance for the use of genetic testing in nephrology practice.
