# Supplementary material for: Genetic Testing for Chronic Kidney Diseases: Clinical Utility and Barriers Perceived by Nephrologists
Source: Kidney Med. 2021 Oct 5;3(6):1050–6. doi: 10.1016/j.xkme.2021.08.006 (PMC8664736; doi:10.1016/j.xkme.2021.08.006)
Supplement: Supplementary File (PDF) — Figure S1; Tables S1-S3. [file mmc1.pdf]

Figure S1: Genetic Test ordering trends among U.S. nephrologists stratified by Region and Institution type

Reported number of tests ordered per month were stratified by the either US Region (specified in Table S1, or by Institution type) as indicated by each respondent (n= 149). Differences in mean number of tests were not significant (p> 0.05) for all comparisons as determined by Kruskal-Wallis Test.

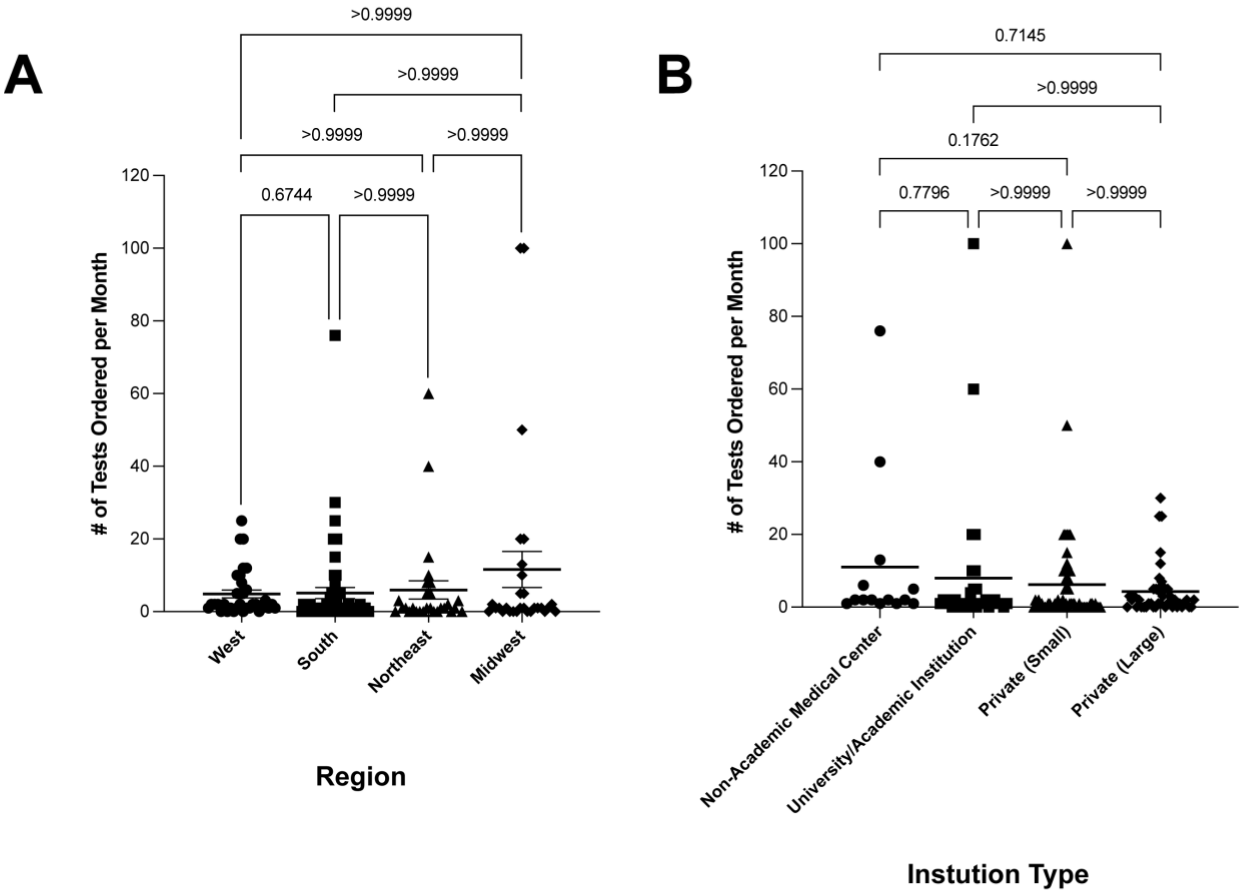

Table S1: Survey Questions

*** Screening Question:** Survey was terminated if any of the following criteria were met:
(1) Specialty = transplant nephrology, pediatric, surgery, **(2)** Not board certified, **(3)** Practicing less than 2 years.

**All Respondents**	**N = 149**	**(%)**
**1. What is your affiliation?**		
University/academic institution	32	(21.5)
Non-academic medical center	14	(9.4)
Private practice, greater than 10 providers	43	(28.9)
Private practice, less than 10 providers	60	(40.3)
**2. What is your specialty?***		
General nephrology	149	(100)
Transplant nephrology	--	--
Pediatric nephrology	--	--
Surgery	--	--
Other	--	--
**3. Are you board certified in general nephrology?***	149	(100)
**4. How long have you been practicing Nephrology?***		
< 2 years	--	--
≥ 2 years and < 5 years	5	(3.3)
≥ 5 years and < 10 years	25	(16.8)
≥ 10 years and < 20 years	69	(46.3)
≥ 20 years and < 30 years	39	(26.2)
≥ 30 years	11	(7.4)
**5. In which state do you currently practice?**		
Alabama (South)	2	(1.3)
Arizona (West)	3	(2.0)
Arkansas (South)	3	(2.0)
California (West)	22	(14.8)

Connecticut (Northeast)	1	(0.7)
Delaware (South)	2	(1.3)
District of Columbia (South)	1	(0.7)
Florida (South)	10	(6.7)
Georgia (South	3	(2.0)
Illinois (Midwest)	7	(4.7)
Indiana (Midwest)	5	(3.4)
Kansas (Midwest)	1	(0.7)
Louisiana (South)	3	(2.0)
Maryland (south)	2	(1.3)
Massachusetts (Northeast)	3	(2.0)
Michigan (Midwest)	3	(2.0)
Minnesota (Midwest)	2	(1.3)
Mississippi (south)	3	(2.0)
Nebraska (Midwest)	1	(0.7)
Nevada (West)	2	(1.3)
New Jersey (Northeast)	8	(5.4)
New York (Northeast)	5	(3.4)
North Carolina (South)	9	(6.0)
Ohio (Midwest)	9	(6.0)
Oklahoma (South)	1	(0.7)
Oregon (West)	3	(2.0)
Pennsylvania (Northeast)	10	(6.7)
Rhode Island (Northeast)	1	(0.7)
South Carolina (South)	3	(2.0)
Tennessee (South)	4	(2.7)
Texas (South)	9	(6.0)
Utah (West)	2	(1.3)
Virginia (South)	3	(2.0)
Washington (West)	2	(1.3)
Wisconsin (Midwest)	1	(0.7)

**6. Who typically refers patients to your office?**		
Primary care physician	149	(100)
Other:	--	--
**7. How much education on genetics did you receive during your nephrology training?**		
None	10	(6.7)
Limited	108	(72.5)
Extensive	31	(20.8)
**8. Have your patients asked you about genetic testing?**		
Yes, my patients often ask	13	(8.7)
Yes, but not frequently	95	(63.8)
No, my patients have never asked	41	(27.5)
**9. How many unique general nephrology patients do you see in one month?**		
Provide a numerical value	Mean (±SD)	
Total responses: 149	269.7 (± 136.7)	
**10. What % of your patients are in the below stages when they are first referred to you?**		
Provide a numerical value for each category (Total responses: 149)	Mean (±SD)	
Stage 1:	5.4 (± 4.4)	
Stage 2:	9.1 (± 5.8)	
Stage 3a:	22.5 (± 9.6)	
Stage 3b:	25.7 (± 8.0)	
Stage 4:	20.7 (± 9.3)	
Stage 5:	8.6 (± 5.1)	
Stage 6/ ESRD:	8.1 (± 8.8)	
**11. Approximately how many genetic tests do you order on average every month?**		
Provide a numerical value	Mean (±SD)	
Total responses: 149	6.5 (± 15.3)	
**12. Among the genetic tests you order every month, how many on average are for *APOL1?***		
Provide a numerical value	Mean (±SD)	
Total responses: 149	4.3 (± 9.3)	
**For those who have ordered genetic tests (Users)**	**N = 107**	**(%)**

**13. What type of genetic testing do you typically order on your adult CKD patients?**		
Small, targeted panel based on clinical symptoms (e.g. cystic, glomerular nephrolithiasis)	53	(49.5)
Expanded panel or full exome to cover all types of disease	11	(10.3)
A combination of small and expanded panels, depending on the patient	43	(40.2)
**14. What % of your adult CKD population has a diagnosis confirmed by genetic testing?**		
Less than 1%	36	(33.6)
2-5%	37	(34.6)
6-10%	15	(14.0)
11-25%	13	(12.1)
26-50%	5	(4.7)
>50%	1	(0.9)
**15. Have the results of a genetic test changed how you manage your patient?**		
Yes, often	18	(16.8)
Yes, sometimes	62	(57.9)
Yes, but rarely	18	(16.8)
No, clinical management would not change	9	(8.4)
**16. In which clinical scenarios do you believe genetic testing has the most value?** *(can select more than 1 option)*		
All patients with CKD	12	(11.2)
Patients with unknown etiology of CKD	74	(69.2)
Specific clinical diagnoses	80	(74.8)
Pediatric patients	27	(25.2)
**16a. Which of the specific clinical diagnoses do you believe genetic testing has the most value?** *(can select more than 1 option)*		
Cystic	74	(69.2)
Glomerular	60	(56.1)
Electrolyte abnormalities	39	(36.4)
Nephrolithiasis	33	(30.8)
CAKUT	29	(27.1)
Hypertension	14	(13.1)

Diabetic nephropathy			8		(7.5)
Tubulointerstitial disease			38		(35.5)
Other (specify)			1 (TMA)		(0.9)
**17. How would you rate each of the following as barriers to ordering genetic testing?**					
Use a scale from 1 (Not a Barrier) to 5 (Significant Barrier).	**1**	**2**	**3**	**4**	**5**
Lack of proven clinical utility	3	22	36	35	11
Concern about cost to the patient	3	4	15	36	49
Availability/ease of testing	7	7	28	30	35
Lack of knowledge on interpreting the results	8	25	31	34	9
Lack of access to genetics experts to assist in ordering and results interpretation	13	15	24	35	20
Other:	1	1	5	2	--
**18. How important is each type of support to make it easier to order genetic testing for your patients?**					
Use a scale from 1 (Not Important) to 5 (Extremely Important).	**1**	**2**	**3**	**4**	**5**
Access to genetic counselors for myself and my patient(s)	3	5	21	50	28
Easy ordering process	1	3	17	42	44
Detailed results with implications for the patient and/or their biologica relatives	--	2	18	38	49
Insurance/billing support	--	4	16	41	46
Other:	--	--	--	2	1

**For those who have NOT ordered genetic tests (non-users)**	**N = 42**	**(%)**
**19. In which clinical scenarios do you believe genetic testing has the most value?** *(can select more than 1 option)*		
All patients with CKD	--	--
Patients with unknown etiology of CKD	16	(38.1)
Specific clinical diagnoses	36	(85.7)
Pediatric patients	13	(30.9)
I don't see value in genetic testing	2	(4.8)
**19a. Which of the specific clinical diagnoses do you believe genetic testing has the most value?** *(can select more than 1 option)*		

Cystic	32	(76.2)
Glomerular	22	(52.4)
Electrolyte abnormalities	20	(47.6)
Nephrolithiasis	9	(21.4)
CAKUT	6	(14.3)
Hypertension	4	(9.5)
Diabetic nephropathy	--	--
Tubulointerstitial disease	8	(19.0)
Other (specify)	--	--

**20. How would you rate each of the following as barriers to ordering genetic testing?** *Use a scale from 1 (Not a Barrier) to 5 (Significant Barrier).*	**1**	**2**	**3**	**4**	**5**
Lack of proven clinical utility	6	3	10	17	6
Concern about cost to the patient	1	4	2	6	29
Availability/ease of testing	1	1	3	13	24
Lack of knowledge on interpreting the results	3	10	9	14	6
Lack of access to genetics experts to assist in ordering and results interpretation	3	3	9	18	9
Other:	2	--	1	1	2

**20a. How important would each type of support be to make it easier to order genetic testing for your patients?** *Use a scale from 1 (Not Important) to 5 (Extremely Important).*	**1**	**2**	**3**	**4**	**5**
Access to genetic counselors for myself and my patient(s)	1	2	4	12	23
Easy ordering process	--	--	3	14	25
Detailed results with implications for the patient and/or their biologica relatives	--	--	3	10	29
Insurance/billing support	--	--	4	9	29
Other:	1	--	--	--	--

**All Respondents**	**N = 149**	**(%)**
**21. What education would you like to receive on genetic testing and CKD?** *(can select more than 1 option)*		
I need a refresher on genetic causes of CKD.	100	(67.1)

I need to know how to talk to my patients about the options for testing an the result implications.	81	(54.4)
Resources to help educate my patients on genetic causes of CKD and family planning needs	103	(69.1)
None	5	(3.4)
**22. What kinds of patient education about genetic kidney disease would you find most useful?** *(can select more than 1 option)*		
Basics of genetic kidney disease	107	(71.8)
Implications for family; such as living related donors, family planning	126	(84.6)
Details on how to interpret results of genetic tests	111	(74.5)
Privacy of genetic information	66	(44.3)
Insurance implications of a positive finding	89	(59.7)

Table S2: Genetic Testing Use Patterns in Survey Respondents

**Institution Type**	**N (%)**	**Users (N=107)**	**Non-Users (N=42)**
University/Academic Institution	32 (21.5)	25 (23.4)	7 (16.7)
Non-academic medical center	14 (9.4)	14 (13.1)	0 (0.0)
Large private practice (> 10 providers)	43 (28.8)	30 (28.0)	13 (31.0)
Small private practice (< 10 providers)	60 (40.3)	38 (35.5)	22 (52.4)
**Region**			
Midwest	29 (19.5)	20 (18.7)	9 (21.4)
Northeast	28 (18.8)	19 (17.8)	9 (21.4)
South	58 (38.9)	39 (36.4)	19 (45.2)
West	34 (22.8)	29 (27.12)	5 (12.0)
**Genetics Education**			
None	10 (6.7)	2 (1.9)	8 (19.0)
Limited	108 (72.5)	75 (70.1)	33 (78.6)
Extensive	31 (20.8)	30 (28.0)	1 (2.4)

Table S3. Perceived barriers to genetic testing (Genetics Education Level)

	Users (N=107)			Non-Users (N=42)		
	None	Limited	Extensive	None	Limited	Extensive
	2 (1.9)	75 (70.1)	30 (28.0)	8 (19.0)	33 (78.6)	1 (2.4%)
**Barriers**	**N (%)**					
cost of testing	2 (100.0)	38 (50.7)	9 (30.0)	4 (50.0)	25 (75.8)	0 (0.0)
availability/ease of testing	1 (50.0)	26 (34.7)	8 (26.7)	4 (50.0)	20 (60.6)	0 (0.0)
access to genetics experts	1 (50.0)	15 (20.0)	4 (13.3)	2 (25.0)	7 (21.2)	0 (0.0)
lack of proven clinical utility	0 (0.0)	8 (10.7)	3 (10.0)	2 (25.0)	4 (12.1)	0 (0.0)
knowledge about result interpretation	1 (50.0)	7 (9.3)	1 (3.3)	2 (25.0)	4 (12.1)	0 (0.0)
